# Depalmitoylation by Palmitoyl-Protein Thioesterase 1 in Neuronal Health and Degeneration

**DOI:** 10.3389/fnsyn.2019.00025

**Published:** 2019-08-29

**Authors:** Kevin P. Koster, Akira Yoshii

**Affiliations:** ^1^Department of Anatomy and Cell Biology, University of Illinois at Chicago, Chicago, IL, United States; ^2^Department of Pediatrics, University of Illinois at Chicago, Chicago, IL, United States; ^3^Department of Neurology, University of Illinois at Chicago, Chicago, IL, United States

**Keywords:** palmitoylation, depalmitoylation, PPT1, lipofuscinosis, NMDA, neurodegeneration

## Abstract

Protein palmitoylation is the post-translational, reversible addition of a 16-carbon fatty acid, palmitate, to proteins. Protein palmitoylation has recently garnered much attention, as it robustly modifies the localization and function of canonical signaling molecules and receptors. Protein depalmitoylation, on the other hand, is the process by which palmitic acid is removed from modified proteins and contributes, therefore, comparably to palmitoylated-protein dynamics. Palmitoylated proteins also require depalmitoylation prior to lysosomal degradation, demonstrating the significance of this process in protein sorting and turnover. Palmitoylation and depalmitoylation serve as particularly crucial regulators of protein function in neurons, where a specialized molecular architecture and cholesterol-rich membrane microdomains contribute to synaptic transmission. Three classes of depalmitoylating enzymes are currently recognized, the acyl protein thioesterases, α/β hydrolase domain-containing 17 proteins (ABHD17s), and the palmitoyl-protein thioesterases (PPTs). However, a clear picture of depalmitoylation has not yet emerged, in part because the enzyme-substrate relationships and specific functions of depalmitoylation are only beginning to be uncovered. Further, despite the finding that loss-of-function mutations affecting palmitoyl-protein thioesterase 1 (PPT1) function cause a severe pediatric neurodegenerative disease, the role of PPT1 as a depalmitoylase has attracted relatively little attention. Understanding the role of depalmitoylation by PPT1 in neuronal function is a fertile area for ongoing basic science and translational research that may have broader therapeutic implications for neurodegeneration. Here, we will briefly introduce the rapidly growing field surrounding protein palmitoylation and depalmitoylation, then will focus on the role of PPT1 in development, health, and neurological disease.

## Introduction

Protein palmitoylation is the post-translational covalent linkage of palmitic acid (palmitate) to, typically, cysteine residues on target proteins (S-palmitoylation). This modification is a form of protein acylation, which adds a fatty acid to proteins and increases their hydrophobicity, facilitating their interaction with lipid membranes. In contrast to other modes of protein acylation (e.g., myristoylation, farnesylation), palmitoylation is generally reversible. Therefore, palmitoylation can effectively act as a post-translational “switch” on some proteins, similarly to phosphorylation, and provide dynamic control over protein localization or function. Indeed, palmitoylation plays critical roles in protein trafficking and strongly influences the stability of proteins (see Dunphy and Linder, 1998; Linder and Deschenes, 2007; Fukata and Fukata, 2010; Salaun et al., 2010; Chamberlain et al., 2013; Montersino and Thomas, 2015).

The prevalence of palmitoylation in neurons and its specific role in the synaptic targeting of some proteins (e.g., PSD-95) support the notion that palmitoylation is particularly adapted to regulate synaptic protein function. Indeed, vesicular release proteins, neurotransmitter receptor subunits, key synaptic scaffolds, and signaling proteins are regulated by palmitoylation (Kang et al., 2008). It is not surprising that disrupted palmitoylation of proteins central to adult-onset neurodegenerative diseases, such as Alzheimer’s disease, is emerging as an important pathogenic mechanism in these disorders (Cho and Park, 2016). In line with this notion, mutations in the enzymes responsible for palmitoylation and depalmitoylation often lead to neurological diseases (Zaręba-Kozioł et al., 2018).

Palmitoylation is executed by protein palmitoyltransferases (PATs), enzymes containing a zinc finger domain with a conserved DHHC motif and, accordingly, called ZDHHCs (Fukata et al., 2004; Lemonidis et al., 2015). These PATs largely act in the endoplasmic reticulum and Golgi apparatus, though palmitoylation also occurs on endo-lysosomes and at the plasma membrane (Ohno et al., 2006; Noritake et al., 2009; Korycka et al., 2012).

As protein palmitoylation is a reversible process, both the attachment and cleavage of palmitic acid are critical for regulating the amounts and localization of modified proteins. The rates of palmitoylation and depalmitoylation can vary widely between protein type; that is, while some proteins undergo multiple cycles of palmitoylation and depalmitoylation throughout their lifetime, others are depalmitoylated only once. Nevertheless, appropriate expression and function of each substrate requires a proper balance between the two processes and, notably, palmitic acid must be removed prior to degradation of modified proteins in the lysosome.

Protein thioesterases, or depalmitoylases, mediate the depalmitoylation of modified proteins, thereby completing a cycle of this reversible post-translational modification. Currently, three classes of depalmitoylases have been identified; the acyl-protein thioesterases (APTs), the α/β hydrolase domain-containing 17 proteins (ABHD17s), and the palmitoyl-protein thioesterases (PPTs). The APTs shuttle between the Golgi and cytosol to depalmitoylate their substrates (Vartak et al., 2014) and have recently attracted attention in the cancer field for their activity toward Ras, the oncogenic small GTPase (Dekker et al., 2010; Xu et al., 2012). The ABDH17s were recently discovered and also depalmitoylate Ras (Lin and Conibear, 2015), though further research has focused on the activity of ABHD17s toward neuronal or synaptic proteins (Yokoi et al., 2016; Tortosa et al., 2017).

Palmitoyl-protein thioesterase 1 (PPT1) facilitates the morphological development of neurons, synaptic function in mature cells, and is the first depalmitoylating enzyme to be linked to a genetic disorder. Specifically, mutations that disrupt PPT1 function cause the devastating neurodegenerative disease, infantile neuronal ceroid lipofuscinosis, emphasizing the importance of depalmitoylation in neuronal health (Vesa et al., 1995). Despite its significance, the mechanistic understanding of PPT1-mediated depalmitoylation has progressed relatively slowly. Thus, this mini review article focuses on the recent advances made in the understanding of PPT1 function in health and disease to emphasize the crucial role played by PPT1 as a depalmitoylase.

## Palmitoyl-Protein Thioesterase 1 (PPT1) Is a Regulator of the Autophagy-Lysosome Pathway

In peripheral cell types, PPT1 is primarily sorted to the lysosome through the classical mannose-6-phoshate pathway, where it serves the role as one of approximately 60 lysosomal hydrolases (Verkruyse and Hofmann, 1996). Interestingly, PPT1 may exhibit an auxiliary pH optimum near pH = 7, distinguishing PPT1 from most lysosomal enzymes which function within the acidic pH of the lysosome (pH 4.5–5; Camp and Hofmann, 1993; Verkruyse and Hofmann, 1996; Cho and Dawson, 2000). Lysosomal enzymes are involved in protein degradation and PPT1 is no exception—PPT1 depalmitoylates proteins in the lysosome prior to their degradation.

**Figure 1:**
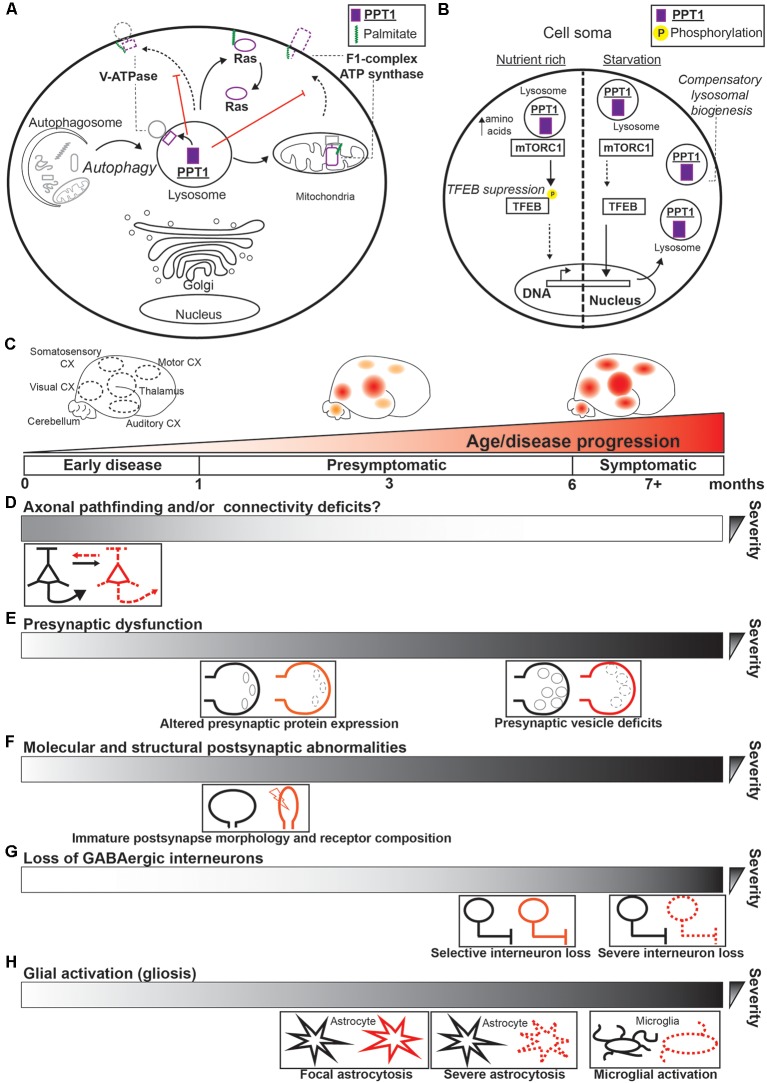
Palmitoyl-protein thioesterase 1 (PPT1) functions and dysfunction in infantile NCL (CLN1). **(A)** Substrates of PPT1 in peripheral cell types are depicted in their respective subcellular localization within the cell soma. Autophagy (including mitophagy) is depicted as flowing into the lysosome, where PPT1 depalmitoylates and contributes to the degradation of exhausted macromolecules. Arrows indicate depalmitoylation by PPT1 or the flow of materials into the lysosome. Red lines indicate the effect of PPT1-mediated depalmitoylation on localization of proteins, where the dotted lines represent reduced trafficking to one or another site. Both the vacuolar ATPase (V-ATPase; V0a1 subunit) and F1 complex of ATP synthase mislocalize to the plasma membrane in the absence of depalmitoylation by PPT1. **(B)** Example of the role of PPT1 in one well-defined mechanism of bioenergetic homeostasis. *Left*, under nutrient-rich conditions, TFEB is phosphorylated by mammalian target of rapamycin complex 1 (mTORC1) and does not enter the nucleus. *Right*, during starvation, mTORC1 disassociates from the lysosomal interface and cannot phosphorylate TFEB, which enters the nucleus to increases transcription of lysosomal biogenesis proteins and PPT1 (Jegga et al., 2011; Settembre et al., 2012). **(C–H)** Summary of the systems disrupted by loss of PPT1 that contribute to CLN1 progression. **(C)** Schematic demonstrating the spatiotemporal pattern of CLN1 disease pathology, which progresses in the characteristic pattern: thalamus > visual cortex > sensory cortices ≥ motor cortex (Kielar et al., 2007). The timeline of disease progression is also depicted in terms of disease phase (e.g., presymptomatic) and age of *Ppt1^−/−^* mice (in months). Gradients in **(D–H)** depict the relative timing of each pathology described. **(D)** In the earliest presymptomatic phase (i.e., *in utero* and neonatal), axonal and neurite connectivity is disrupted (Chu-LaGraff et al., 2010; Lange et al., 2018), potentially leading to dysfunctional circuit formation. **(E)** As early as 1-month, *Ppt1*^−/−^ mice demonstrate reduced expression of critical pre-synaptic proteins (e.g., SNAP25) and abnormal expression of proteins associated with protection from Wallerian degeneration (Kielar et al., 2009). At 6-months, reductions in synaptic vesicle pool size are reported (Kim et al., 2008). **(F)** As early as postnatal day 33 (~1 month) structural and molecular defects of the postsynapse are detected in *Ppt1*^−/−^ visual cortex that contributes to excitotoxicity (Koster et al., 2019). **(G)** Select GABAergic neuron populations begin to degenerate in the thalamus and cortex by 5 months (Kielar et al., 2007). These interneurons undergo robust degeneration by 7-months, preceding the onset of seizure (Kielar et al., 2007). **(H)** Beginning in the presymptomatic phase, significant astrocytosis is detected at 3-months in the thalamus, cortex, spinal cord, and cerebellum of *Ppt1*^−/−^ mice. Astrocytosis becomes severe in the thalamus and robust in cortical regions by 5 months. At 7-months, astrocytosis is widespread and severe, while focal microglial activation is detected in both thalamus and cortex (Bible et al., 2004; Kielar et al., 2007; Macauley et al., 2009; Shyng et al., 2017).

PPT1 was identified as the first depalmitoylating enzyme by monitoring the enzymatic removal of [^3^H]palmitate from H-Ras (Camp and Hofmann, 1993; Cho and Dawson, 2000) and was subsequently cloned (Camp et al., 1994). In addition, the F_1_-complex of the mitochondrial ATP synthase is a* bona fide* PPT1 substrate and its depalmitoylation is required proper localization of the complex (Lyly et al., 2008; Figure 1B). Recent studies in *Drosophila* have identified proteins involved in endocytosis, exocytosis, and endo-lysosomal trafficking as PPT1 interactors (Buff et al., 2007; Saja et al., 2010) and *in vitro* experiments demonstrate that endocytosis is disrupted in PPT1-null human fibroblasts (Ahtiainen et al., 2006). These data demonstrate specific roles for PPT1-mediated depalmitoylation in the regulation of signaling proteins, transmembrane receptors, and other molecules.

A major source of PPT1 substrates entering the lysosome is from macroautophagy (referred to hereafter as autophagy). Autophagy is a conserved cellular system by which cytoplasmic constituents are engulfed in dual membrane autophagosomes and trafficked to lysosomes for degradation (Mizushima, 2007; Mizushima and Komatsu, 2011). This process encourages cellular quality control mechanisms, remodeling of the local environment, and allows for the recycling of biological building blocks when resources are limited (Mizushima and Komatsu, 2011). Being the terminus of this and other degradative pathways, the lysosome senses the cellular nutritional state and serves as a signaling interface governing anabolic and catabolic process in the cell (Perera and Zoncu, 2016). Importantly, PPT1 is one of several target proteins upregulated by transcription factor EB (TFEB), the master regulator of lysosome and autophagosome biogenesis (Jegga et al., 2011).

In this mechanism crucial for cellular bioenergetics, amino acid starvation is directly recognized by the lysosomal vacuolar ATPase (V-ATPase) and, in conjunction with mammalian target of rapamycin complex 1 (mTORC1) signaling, triggers the nuclear translocation of TFEB (Settembre et al., 2011, 2012; Zoncu et al., 2011; Figure 1A). This causes a compensatory upregulation in autophagosomal and lysosomal biogenesis, as well as PPT1 expression that accelerates degradation of cellular materials for recycling (Settembre et al., 2011, 2012; Figure 1A). Interestingly, PPT1 depalmitoylates the V-ATPase component, V0a1 (Bagh et al., 2017); hence, increased palmitoylation of V0a1 in PPT1-null cells causes its mislocalization to the plasma membrane by enhanced interactions with the adaptor protein AP-2 (Bagh et al., 2017; Figure 1B). Moreover, inhibiting PPT1 activity suppresses V-ATPase function and, consequently, autophagy (Rebecca et al., 2017). These data imply PPT1 plays an active role in tuning the level of cellular autophagy through interactions with the V-ATPase. Taken together, one function of PPT1, particularly in peripheral cell types, involves the coordinated depalmitoylation and degradation of proteins through the autophagy-lysosome pathway (Figures 1A,B).

## CLN1 Is Caused by Loss-of-Function Mutations in PPT1

The neuronal ceroid lipofuscinoses (NCLs) are a class of systemic diseases principally defined by fatal neurodegeneration, manifested by vision loss (blindness), seizures, psychomotor deterioration, and premature death. Mutations in the Infantile NCL (*CLN1*) gene, which encodes PPT1, cause the infantile form of NCL. CLN1 is particularly devastating, with a disease onset of between 6 and 24 months. Afflicted patients present with regression of developmental milestones, myoclonic seizures, blindness, and death before the age of 10 (Nita et al., 2016).

Neuropathologically, CLN1 is defined by the accumulation of storage material, termed lipofuscin or autofluorescent lipopigment, within the lysosomes of neurons. These inclusion bodies correspond to granular osmiophilic deposits (GRODs), also lysosomal accumulations, observed using electron microscopy in virtually every organ tissue of afflicted patients (Nita et al., 2016). Hence, CLN1 is further categorized as a lysosomal storage disorder. However, whether lipofuscin is directly neurotoxic remains controversial, with most evidence demonstrating that it is not directly related to neuronal dysfunction (see Palmer et al., 2013; Cooper et al., 2015).

In 2001, the first mouse model of CLN1 was generated (*Ppt1^−/−^*), which largely recapitulates the human disease phenotype (Gupta et al., 2001). Since, a body of work has described the accumulation of storage material, neuronal apoptosis, and behavioral phenotype in this (Gupta et al., 2001; Cooper et al., 2002; Bible et al., 2004; Kielar et al., 2007, 2009; Kim et al., 2008; Macauley et al., 2009; Dearborn et al., 2015) and alternative disease models (Bouchelion et al., 2014). These studies demonstrate that CLN1 pathology progresses in a characteristic anatomical pattern, arising and becoming robust in the thalamus before spreading to primary sensory cortices, particularly the visual cortex, and then to remaining cortical regions, at which point pathology is severe and symptoms emerge (Figure 1C). Collectively, this work provides a landscape of disease pathology necessary for measuring the efficacy of prospective therapeutics. Still, the precise mechanisms underlying these pathologies, and the weight of each heretofore discovered mechanism on the disease progression remain uncertain (Figures 1D–H).

PPT1 depalmitoylates molecules important for axon outgrowth and neurite extension (see below). Furthermore, recent data (albeit in *Ppt1^−/−^* *Drosophila*) demonstrate that lack of PPT1 causes widespread axonal pathfinding deficits and dendritic morphological defects (Chu-LaGraff et al., 2010). Experiments *in vitro* support these findings, as primary *Ppt1*^−/−^ neurons show poor axonal (i.e., putative axons) extension compared to wild type cells (Lange et al., 2018). These data suggest that the pathological cascade of CLN1 may begin as early as *in utero*, with abnormal neuronal circuit formation potentially contributing to subsequent dysfunction (Figure 1D).

Pre-synaptic protein levels (e.g., SNAP25) are decreased as early as 3-months in the thalamus of *Ppt1^−/−^* mice and, intriguingly, proteins associated with the slowing of Wallerian degeneration (Wishart et al., 2007) show abnormal expression even earlier (1 month; Kielar et al., 2009; Figure 1E). Also, evoked pre-synaptic vesicle release is dampened in *Ppt1*^−/−^ primary neurons (Virmani et al., 2005). Furthermore, *Ppt1*^−/−^ mice demonstrate reduced pre-synaptic vesicle pool size by 6-months of age in the cortex (Kim et al., 2008; Figure 1E). Therefore, it is plausible that dysregulated vesicle recycling and the consequent decrease in evoked synaptic transmission may lead to an axonal “dying back” pathology that contributes to other neurodegenerative disorders (Brady and Morfini, 2010; Kanaan et al., 2013).

Recent work in *Ppt1^−/−^* mice is beginning to shed light on post-synaptic deficits contributing to disease pathogenesis (Finn et al., 2012; Koster et al., 2019; Sapir et al., 2019). For instance, in both dissociated neuronal cultures and juvenile mouse visual cortex, PPT1-null neurons demonstrate immature dendritic spine morphology compared to wild-type neurons (Koster et al., 2019; Sapir et al., 2019). Furthermore, the expression of post-synaptic N-methyl-D-aspartate receptors (NMDARs), which are critical for various forms of synaptic plasticity, is abnormal by postnatal day 33 and persists, at least, until 2 months in the *Ppt1*^−/−^ visual cortex (Koster et al., 2019). These data are corroborated by the finding that *Ppt1*^−/−^ neurons are specifically susceptible to NMDAR-induced excitotoxicity (Finn et al., 2012). Moreover, while putative NMDAR-calcium influxes are largely restricted to individual dendritic spines in wild type neurons, calcium entry at *Ppt1*^−/−^ dendrites is both more robust and diffuse (Koster et al., 2019). These data show that lack of PPT1 leads to increased post-synaptic calcium influx in developing neurons that partially underlies excitotoxic insult in CLN1 (Koster et al., 2019; Figure 1F).

One of the initial findings in *Ppt1^−/−^* mice demonstrated a significant loss of γ-aminobutyric acid (GABA)-ergic interneurons in cortical regions, with more pronounced degeneration compared to pyramidal neurons in some areas and profound loss by 7-months of age (Cooper et al., 2002; Bible et al., 2004; Figure 1G). Notably, significant loss of cortical GABAergic neurons closely precedes seizure in *Ppt1*^−/−^ mice (Kielar et al., 2007), implying that this is a distal, though consequential, cause of disease progression in mice.

Among the best-characterized pathological changes in *Ppt1^−/−^* mice is the inflammatory activation of astrocytes (Kielar et al., 2007). This astrocytosis is detectable as early as 3 months in sensory cortices while it is already pronounced in the thalamus at the same age (Kielar et al., 2007; Figure 1H). All levels of the spinal cord also show glial activation by 3 months (Shyng et al., 2017). Interestingly, astrocytic activation precedes neuronal death within each brain area (Figures 1C–H), implying that loss of PPT1 has even greater effects on glial function compared to neurons and that this may be a proximal event underlying CLN1 progression. Indeed, PPT1 is expressed in all glial cell-types (Kelley et al., 2018) and *Ppt1*^−/−^ astrocytes *in vitro* are activated at baseline, show deficits in calcium handling, and negatively impact neuronal function in co-cultures compared to those from WT animals (Lange et al., 2018). Furthermore, in the cerebellum of *Ppt1*^−/−^ mice, astrocytosis is accompanied by reduced expression of glutamate aspartate transporter 1 (GLAST-1; Macauley et al., 2009). As astrocytes typically buffer neurotransmitter at synapses, in part through GLAST-1, and are important to the development of seizure (Tian et al., 2005; Barker-Haliski and White, 2015), this is one mechanism by which loss of PPT1 causes disease symptoms. Although, glial substrates of PPT1 that underlie this disruption are currently unknown.

## Regulation of Axonal Guidance and Pre-synaptic Mechanisms by PPT1

**Figure 2:**
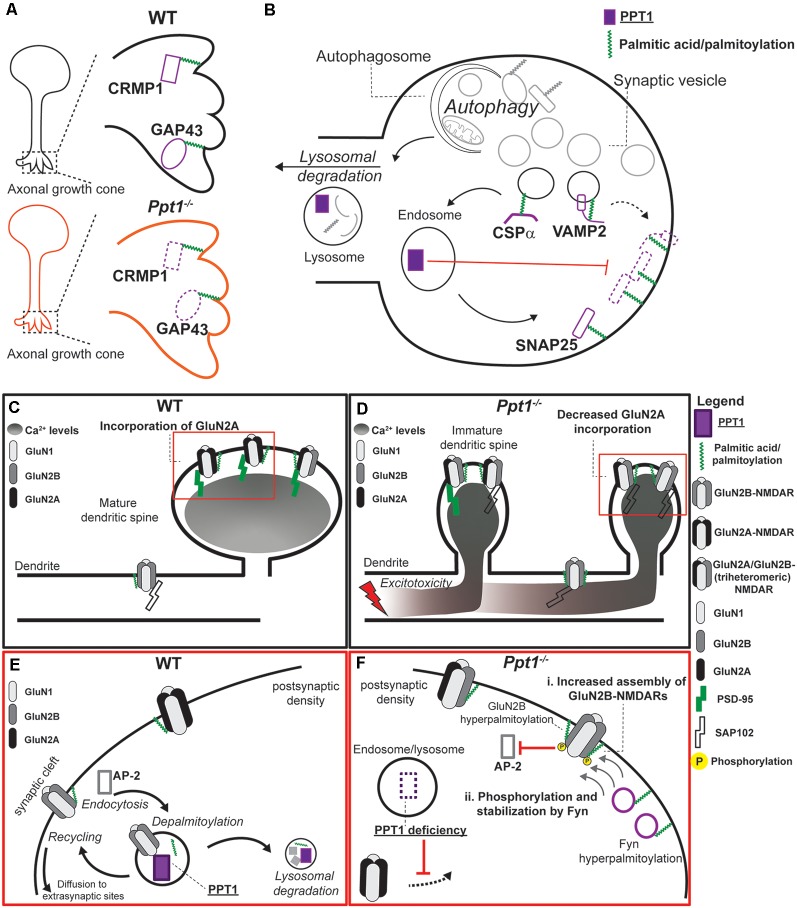
Pre-synaptic and post-synaptic mechanisms regulated by PPT1. **(A)** In developing neurons, PPT1 depalmitoylates GAP43 and potentially collapsin response mediator protein 1 (CRMP1) at axonal growth cones (*insets*). Loss of PPT1 causes disruption or simplification of extending axons, although the mechanism remains unclear. **(B)** At the presynapse, PPT1 contributes to the local depalmitoylation of its substrates as well as to lysosomal degradation of expired proteins. Red lines indicate the effect of PPT1-mediated depalmitoylation on localization of proteins. Both VAMP2 and SNAP25 are trapped in the membrane in the absence of depalmitoylation by PPT1. PPT1 is illustrated in color code with its substrates. Pre-synaptic autophagy mechanisms also feed into the endo-lysosomal pathway (Liang, 2019), though the specific roles of PPT1 therein remain uncharacterized. **(C–F)** Loss of PPT1 causes post-synaptic dysregulation of dendritic spine morphology, NMDAR function, and calcium dynamics. **(C)** In the WT visual cortex, early-life visual experience triggers GluN2A expression and incorporation into synaptic NMDARs that are scaffolded by PSD-95 (Quinlan et al., 1999a,b). This incorporation generally occurs at the post-synaptic density of maturing dendritic spines, while GluN2B receptors are generally further from the synapse (van Zundert et al., 2004). **(D)** In the *Ppt1*^−/−^ visual cortex, structurally immature synapses (filipodia and thin spines) incorporate primarily GluN2B-containing NMDARs and are scaffolded by SAP102. The overrepresentation of GluN2B-containing NMDARs results in higher levels of calcium into the extrasynaptic dendritic areas and induces excitotoxicity. **(E)** Magnification of red square in C. In WT neurons, basal GluN2B and Fyn palmitoylation maintain appropriate stability of GluN2B at the synaptic surface. **(F)** Magnification of red square in D. Loss of *Ppt1*^−/−^ causes hyperpalmitoylation of both GluN2B and Fyn kinase, which leads to at least two potential mechanisms by which lack of PPT1 causes a delayed GluN2 subunit switch and disrupted calcium dynamics. Specifically, GluN2B-NMDARs may be overly stabilized at the synapse in *Ppt1*^−/−^ neurons *via*: (i) hyperpalmitoylation of GluN2B may increase its half-life, promoting enhanced retention and local assembly of GluN2B-containing NMDARs at or near the synapse or (ii) hyperpalmitoylation of Fyn may enhance its local activity at the PSD, phosphorylating and stabilizing GluN2B at the synapse by restricting its AP-2-dependent endocytosis (Prybylowski et al., 2005). Protein names are listed next to their respective symbols/representations except where denoted with a dotted line.

Recent data support a role for PPT1 in axon guidance (Chu-LaGraff et al., 2010) and neurite extension (Lange et al., 2018; Sapir et al., 2019), processes that govern circuit formation during development. PPT1 depalmitoylates GAP43 (Camp and Hofmann, 1993; Cho and Dawson, 2000; Sapir et al., 2019), which must be palmitoylated to traffic to the advancing growth cone and facilitate axon extension (Denny, 2006). Thus, it is plausible that PPT1 regulates axonal growth *via* GAP43 depalmitoylation and loss of this function in CLN1 causes axonal defects (Figure 2A). Alternatively, PPT1 may influence axon guidance *via* the palmitoylated protein collapsin response mediator protein 1 (CRMP1), with which the enzyme likely interacts (Scifo et al., 2015; Pezzini et al., 2017; Figure 2A). Indeed, CRMP1 acts downstream of Semaphorin3A and Reelin signaling to regulate axonal growth cone collapse and neural migration, respectively (Yamashita et al., 2006; Schmidt and Strittmatter, 2007). However, little is known regarding the effect of palmitoylation on CRMP1 function. Notably, the Src family kinase Fyn is a substrate of PPT1 (Scifo et al., 2015; Tikka et al., 2016; Sapir et al., 2019) and is a major effector of CRMP1-related signaling pathways (Arnaud et al., 2003; Morita et al., 2006; Yamashita et al., 2006; Buel et al., 2010; Pezzini et al., 2017). These data indicate that PPT1 regulates axonal pathfinding through multiple protein interactions. Identifying which pathways are dysfunctional in CLN1 represents an important step in understanding disease progression.

In mature neurons, members of the pre-synaptic machinery, in particular, have been identified as PPT1 substrates. Work in both mouse and human tissue suggests SNAP25 and VAMP2 are PPT1 substrates at the presynapse (Figure 2B). Hence, lack of functional PPT1 leads to the persistent membrane association of VAMP2 and SNAP25, thereby impairing vesicle recycling in both mice and humans (Kim et al., 2008; Figure 2B). In interneurons, the GABA-synthesizing enzyme glutamic acid decarboxylase 65 (GAD65) is a suggested substrate of PPT1 (Kim et al., 2008). Importantly, dynamic palmitoylation of GAD65, i.e., both palmitoylation and depalmitoylation, regulate its trafficking and delivery to synapses (Kanaani et al., 2008). Therefore, altered GABA synthesis due to disrupted GAD65 palmitoylation may represent a pathological feature in CLN1 and represents a starting point for future investigation of the role of PPT1 in the health of these cells.

The pre-synaptic chaperone cysteine string protein α (CSPα, *DNAJ5*), which is mutated in adult-onset neuronal ceroid lipofuscinosis (CLN4), is a PPT1 substrate in a cell-free preparation (Nosková et al., 2011; Henderson et al., 2016; Figure 2B). Levels of PPT1 are also drastically increased in brain tissue from CLN4 patients (Henderson et al., 2016). Further, disease-causing mutations that affect the CSPα palmitoylation lead to defects in synaptic vesicle cycling and altered expression of synaptic proteins that parallel the effects of PPT1 mutations (Nosková et al., 2011; Rozas et al., 2012; Henderson et al., 2016), supporting a role for PPT1 at the presynapse that might involve interactions with CSPα. Alternatively, CSPα co-localizes with the lysosomal marker Lamp-1 in primary mouse neurons and post-mortem brain tissue from an early-stage human CLN4 patient exhibits lysosomal pathology (abundant storage material and detectable gliosis) without apparent pre-synaptic deficits (Benitez et al., 2015; Benitez and Sands, 2017). Moreover, the autophagy-lysosome pathway is robustly disrupted in fibroblasts from CLN4 patients (Benitez and Sands, 2017). These findings suggest interactions between PPT1 and CSPα at the lysosome, the disruption of which might be a primary driver of CLN4. Interestingly, PPT1 levels and activity are also robustly increased CLN4 patient fibroblasts (Benitez and Sands, 2017), pointing to a functional relationship between PPT1 and CSPα. Thus, how dysregulation of CSPα caused by PPT1 mutations contributes to axonal degeneration in CLN1 remains unknown; however, alterations in CSPα protein level are not detected in *Ppt1*^−/−^ visual cortex at or before 2-months (Koster, unpublished observations).

## The Role of PPT1 in Post-synaptic Function

In addition to axonal outgrowth, PPT1 also regulates dendritic spine number and morphology (Lange et al., 2018; Koster et al., 2019; Sapir et al., 2019). Indeed, the Semaphorin3A-CRMP1-Fyn signaling pathway also regulates dendritic spine morphology (Morita et al., 2006; Yamashita et al., 2006), indicating that PPT1 may play specialized but overlapping roles in both pre- and post-synaptic neuritogenesis that culminate in immature connectivity in CLN1.

PPT1 also regulates the molecular configuration of the postsynapse. Typically, the GluN2 subunit of NMDARs undergoes a shift during neurodevelopment, in which receptors incorporating the neonatally-expressed GluN2B subunit are partly supplanted by those containing GluN2A (Stocca and Vicini, 1998; Quinlan et al., 1999a,b; Figure 2C). This GluN2 subunit switch coincides with that of the synaptic scaffold, SAP102, for PSD-95 (van Zundert et al., 2004; Elias et al., 2008) and is important for maturation of NMDAR-mediated signals at the postsynapse (Figure 2C). Compared to wild type visual cortex, protein levels of GluN2A and PSD-95 levels are persistently decreased in *Ppt1^−/−^* animals (Figure 2C vs. Figure 2D), while total levels of GluN2B are unperturbed, suggesting an immature NMDAR composition (Figure 2C).

How does PPT1 Affect the post-synaptic characteristics of NMDARs? Notably, both GluN2B and Fyn kinase are hyperpalmitoylated in *Ppt1^−/−^* neurons compared to WT cells (Koster et al., 2019; Figure 2E vs. 2F). Since palmitoylation often increases protein stability (Percherancier et al., 2001; Abrami et al., 2006; Sharma et al., 2008) GluN2B hyperpalmitoylation may lead to enhanced local assembly of GluN2B-containing NMDARs and, thereby, their increased presence at synapses (Figure 2F). Alternatively, Fyn hyperpalmitoylation may enhance its activity at the synaptic membrane (Webb et al., 2000) and, as Fyn phosphorylates GluN2B, increase the surface retention and number of GluN2B-containing NMDARs (Prybylowski et al., 2005; Hayashi et al., 2009; Figure 2E vs. 2F). Thus, a second potential mechanism by which lack of PPT1 disrupts the GluN2 subunit switch involves this Fyn-dependent GluN2B stabilization (Figure 2F). Together, these data suggest that depalmitoylation by PPT1 is involved the developmental switch from GluN2B to GluN2A and that this switch is disrupted in CLN1.

## Therapeutics for CLN1

There are currently no disease-modifying therapies for CLN1, and treatments focus on symptomatic relief (e.g., anticonvulsant medications; Geraets et al., 2016). Approaches to treating CLN1 include substrate reduction therapies, enzyme or gene-replacement therapies, and pharmacological intervention focused on mimicking PPT1 activity. Substrate reduction therapy trials have shown limited success (Gavin et al., 2013; Levin et al., 2014). In preclinical models, PPT1 mimetic treatment partially reverses disease phenotype and extends the lifespan of PPT1-null animals (Sarkar et al., 2013). Enzyme-replacement therapies using recombinant PPT1 and adeno-associated viral delivery of PPT1 have also showed promise in CLN1 mouse models (Griffey et al., 2004; Hu et al., 2012; Roberts et al., 2012), with simultaneous intrathecal and intracranial administration conferring the greatest benefits (Shyng et al., 2017). Furthermore, the FDA recently approved a clinical trial to test ABO-202, an adeno-associated virus-based gene therapy, in human patients with CLN1. However, due to the early onset and rapid degeneration in humans, diagnosis is often not made until pathology is significant. Therefore, early intervention is imperative and treatment protocols that consider multiple approaches (e.g., pharmacological and gene-replacement therapies) are particularly promising.

## Conclusion

In sum, palmitoylation and depalmitoylation are crucial post-translational processes that facilitate the appropriate localization, function, and abundance of many proteins. Depalmitoylation by PPT1 is involved in several cellular processes, including those related to the autophagy-lysosome pathway, but is crucial in neurons, where PPT1 contributes to axonal outgrowth, neurite extension, and dendritic spine morphogenic pathways. In CLN1, accumulating evidence points to glial activation, pre-synaptic vesicle dysfunction, and post-synaptic deficits in NMDAR maturation due to lack of PPT1. It will be important to validate the substrate profile of PPT1, and determine the role of PPT1-mediated depalmitoylation in synaptic transmission and plasticity. These studies will reveal novel therapeutic strategies for CLN1 and may have broader implications for adult-onset neurodegenerative diseases, for which disrupted palmitoylation and depalmitoylation are also features.

## Author Contributions

KK and AY wrote the manuscript.

## Conflict of Interest Statement

The authors declare that the research was conducted in the absence of any commercial or financial relationships that could be construed as a potential conflict of interest.
